# Supplementary material for: Methionine adenosyltransferase 1a antisense oligonucleotides activate the liver-brown adipose tissue axis preventing obesity and associated hepatosteatosis
Source: Nat Commun. 2022 Mar 1;13:1096. doi: 10.1038/s41467-022-28749-z (PMC8888704; doi:10.1038/s41467-022-28749-z)
Supplement: Supplementary file 1 — Supplementary Information [file 41467_2022_28749_MOESM1_ESM.pdf]

Supplemental Information

Methionine adenosyltransferase 1a antisense oligonucleotides activate the liver-brown adipose tissue axis preventing obesity and associated hepatosteatosis

Diego Sáenz de Urturi[1], Xabier Buqué[1,2], Begoña Porteiro[3], Cintia Folgueira[4], Alfonso Mora[4], Teresa C. Delgado[5], Endika Prieto-Fernández[6], Paula Olaizola[7], Beatriz Gómez-Santos[1], Maider Apodaka-Biguri[1], Francisco González-Romero[1], Ane Nieva-Zuluaga[1], Mikel Ruiz de Gauna[1], Naroa Goikoetxea-Usandizaga[5], Juan Luis García-Rodríguez[1], Virginia Gutierrez de Juan[5], Igor Aurrekoetxea[1,2], Valle Montalvo-Romeral[4], Eva M. Novoa[3], Idoia Martín-Guerrero[6], Marta Varela-Rey[5,8,9], Sanjay Bhanot[10], Richard Lee[10], Jesus M Banales[7,8,11,12], Wing-Kin Syn[1,13,14], Guadalupe Sabio[4], María L. Martínez-Chantar[5,8], Rubén Nogueiras[3,15,16], Patricia Aspichueta[1,2,8*].

[1]Department of Physiology, Faculty of Medicine and Nursing, University of the Basque Country UPV/EHU, Leioa, Spain.

[2]Biocruces Bizkaia Health Research Institute, Barakaldo, Spain

[3]Department of Physiology, CIMUS, University of Santiago de Compostela-Instituto de Investigación Sanitaria, Santiago de Compostela, Spain.

[4]Myocardial Pathophysiology, Centro Nacional de Investigaciones Cardiovasculares (CNIC), Madrid, Spain.

[5]Liver Disease Laboratory, CIC bioGUNE-BRTA (Basque Research & Technology Alliance), Derio, Spain.

[6]Department of Genetics, Physical Anthropology and Animal Physiology, Faculty of Science and Technology, University of the Basque Country UPV/EHU, Leioa, Spain.

[7]Department of Liver and Gastrointestinal Diseases, Biodonostia Health Research Institute - Donostia University Hospital, University of the Basque Country (UPV/EHU), San Sebastian, Spain.

[8]National Institute for the Study of Liver and Gastrointestinal Diseases (CIBERehd, Instituto de Salud Carlos III), Madrid, Spain.

[9]Gene Regulatory Control in Disease Laboratory, CIMUS, University of Santiago de Compostela-Instituto de Investigación Sanitaria, Santiago de Compostela, Spain

[10]IONIS Pharmaceuticals, Carlsbad, California, USA.

[11]Ikerbasque, Basque Foundation for Science, Bilbao, Spain.

[12]Department of Biochemistry and Genetics, School of Sciences, University of Navarra, Pamplona, Spain.

[13]Section of Gastroenterology, Ralph H Johnson, VAMC, Charleston, USA

[14]Division of Gastroenterology and Hepatology, Medical University of South Carolina, Charleston, USA

[15]CIBER Fisiopatología de la Obesidad y Nutrición (CIBERobn), Santiago de Compostela, Spain.

[16]Galician Agency of Investigation, Xunta de Galicia, Spain.

*Correspondence: Patricia Aspichueta, Department of Physiology, Faculty of Medicine and Nursing, University of the Basque Country UPV/EHU, Barrio Sarriena s/n, 48940 Leioa, Spain. Phone: +34 946012896; Fax: +34 946015662; e-mail: patricia.aspichueta@ehu.eus

Inventory of Supplemental Information:

1. Supplemental Tables
2. Supplemental Figures and Figure legends
3. Separate Source data file

Supplementary Table 1.- Corporal and hepatic and renal function parameters in ASO-treated mice.

		CD	HFD	HFD
Parameter	Normal Range	Control ASO (n=5)	Control ASO (n=5)	*Mat1a* ASO 2 (n=5)
ALB (g/l)	26 - 54	32.8 ± 1.4	31.0 ± 1.7	30.9 ± 2.3
ALT (IU/l)	22 - 133	21.2 ± 3.9	45.2 ± 35.4	33.0 ± 14.6
AST (IU/l)	46 – 221	39.4 ± 7.1	54.6 ± 23.4	48.2 ± 12.0
TBIL (mg/dl)	0.1 - 0.7	0.05 ± 0.02	0.03 ± 0.02	0.04 ± 0.01
CRE (mg/dl)	0.1 - 1.8	0.23 ± 0.04	0.21 ± 0.03	0.18 ± 0.03
Urea (mg/dl)	4.3 - 153.9	47.4 ± 12.6	51.2 ± 4.5	50.0 ± 8.2
Body Weight (g)	-	30.3 ± 2.1	45.3 ± 2.8$^{###}$	36.7 ± 2.8**
Liver (g)	-	1.51 ± 0.25	1.63 ± 0.29	1.28 ± 008*
Kidney (g)	-	0.33 ± 0.04	0.37 ± 0.02	0.31 ± 0.13
Spleen (g)	-	0.10 ± 0.02	0.09 ± 0.01	0.11 ± 0.01

2-month-old C57BL/6J mice were fed a chow diet (CD) or a high-fat diet (HFD) for 10 weeks. During the last 4 weeks mice were treated with *Mat1a* antisense oligonucleotide (ASO) 2 (50 mg/kg/week) (n=5) or with control ASO (50 mg/kg/week) (n=5) until sacrifice. Serum albumin (ALB), alanine aminotransferase (ALT), aspartate aminotransferase (AST), total bilirubin (TBIL), creatinine (CRE) and urea; and body, liver, kidney and spleen weight were measured. Values are presented as means ± SD. Statistically significant differences between groups are indicated by *$p<0.05$, and **$p<0.01$ when comparing *Mat1a* ASO vs. control ASO; and by $^{###}p<0.001$ when comparing CD vs HFD (two tailed Student's test). Source data are provided as a Source data file.

Supplementary Table 2.- Body weight of ASO-treated mice fed a chow diet (CD).

Week	Control ASO CD (n=5) (g)	*Mat1a* ASO CD (n=5) (g)
1	25.8 ± 2.5	25.7 ± 1.4
2	25.0 ± 2.0	25.2 ± 1.3
3	25.0 ± 1.6	25.4 ± 1.5
4	25.8 ± 1.8	26.1 ± 1.2
5	26.3 ± 1.7	26.8 ± 1.5
6	25.9 ± 2.2	26.8 ± 1.6
7	26.3 ± 1.8	27.1 ± 1.5
8	26.4 ± 1.7	27.3 ± 1.5
9	27.1 ± 1.8	27.1 ± 1.4
10	27.2 ± 2.2	26.5 ± 2.2

2-month-old C57BL/6J mice were fed a CD for 10 weeks. During the last 4 weeks mice were treated with *Mat1a* antisense oligonucleotide (ASO) (25 mg/kg/week) (n=5) or with control ASO (25 mg/kg/week) (n=5) until sacrifice. Values are presented as means ± SD of grams (g) of body weight. Source data are provided as a Source data file.

Supplementary Table 3.- Corporal and hepatic and renal function parameters in ASO-treated mice.

		CD	HFD	HFD
Parameter	Normal Range	Control ASO (n=5)	Control ASO (n=5)	*Mat1a* ASO (n=5)
ALB (g/l)	26 - 54	34.6 ± 1.0	30.9 ± 1.2$^{###}$	30.8 ± 1.7
ALT (IU/l)	22 - 133	17.4 ± 2.1	43.8 ± 22.1$^{#}$	55.2 ± 21.3
AST (IU/l)	46 - 221	37.8 ± 2.8	51.0 ± 8.6$^{#}$	114.0 ± 42.3*
TBIL (mg/dl)	0.1 - 0.7	0.06 ± 0.01	0.04 ± 0.01$^{#}$	0.06 ± 0.00*
CRE (mg/dl)	0.1 - 1.8	0.19 ± 0.02	0.19 ± 0.05	0.16 ± 0.03
Urea (mg/dl)	4.3 - 153.9	40.4 ± 3.2	47.0 ± 3.2$^{#}$	57.0 ± 3.0***
Body Weight (g)	-	30.1 ± 1.5	50.5 ± 2.6$^{###}$	29.0 ± 0.8***
Liver (g)	-	1.27 ± 0.11	1.90 ± 0.30$^{##}$	1.50 ± 0.10*
Kidney (g)	-	0.25 ± 0.02	0.34 ± 0.03$^{##}$	0.31 ± 0.02
Spleen (g)	-	0.08 ± 0.01	0.14 ± 0.03$^{##}$	0.11 ± 0.01

2-month-old C57BL/6J mice were fed a chow diet (CD) or a high-fat diet (HFD) for 16 weeks. During the last 9 weeks mice were treated with *Mat1a* antisense oligonucleotide (ASO) (25 mg/kg/week) (n=5) or with control ASO (25 mg/kg/week) (n=5) until sacrifice. Serum albumin (ALB), alanine aminotransferase (ALT), aspartate aminotransferase (AST), total bilirubin (TBIL), creatinine (CRE) and urea; and body, liver, kidney and spleen weight were measured. Values are presented as means ± SD. Statistically significant differences between groups are indicated by *p<0.05, and ***p<0.001 when comparing Mat1a ASO vs. control ASO; and by $^{#}$p<0.05, $^{##}$p<0.01 and $^{###}$p<0.001 when comparing CD vs HFD (two tailed Student's test). Source data are provided as a Source data file.

Supplementary Table 4.- Brown adipose tissue epinephrine levels in ASO-treated mice fed with high fat diet (HFD).

Control ASO HFD (n=7) (pg/mg)	*Mat1a* ASO HFD (n=7) (pg/mg)
200.0 ± 83.7	130.6 ± 24.7

2-month-old C57BL/6J mice were fed a HFD for 10 weeks. During the last 4 weeks mice were treated with *Mat1a* antisense oligonucleotide (ASO) (25 mg/kg/week) (n=7) or with control ASO (25 mg/kg/week) (n=7) until sacrifice. Values are presented as means ± SD of picograms of hormone per milligrams of tissue (pg/mg). Source data are provided as a Source data file.

Supplementary Table 5.- Serum fibroblast growth factor (FGF)21 levels in ASO-treated mice fed a chow diet (CD).

Control ASO CD (n=5) (ng/ml)	*Mat1a* ASO CD (n=5) (ng/ml)
0.29 ± 0.18	1.19 ± 0.42**

2-month-old C57BL/6J mice were fed a CD for 10 weeks. During the last 4 weeks mice were treated with *Mat1a* antisense oligonucleotide (ASO) (25 mg/kg/week) (n=5) or with control ASO (25 mg/kg/week) (n=5) until sacrifice. Values are presented as means ± SD of nanograms of FGF21 per milliliter of serum (ng/ml). Statistically significant differences between groups are indicated by **$p<0.01$ when comparing Mat1a ASO vs. control ASO. Source data are provided as a Source data file.

Supplementary Table 6.- Hepatic lipogenic genes expression in ASO-treated mice fed a high fat diet (HFD).

Genes	Control ASO HFD (n=5-7)	*Mat1a* ASO HFD (n=6)
Acetyl-CoA carboxylase 1 (*Acaca*)	1.00 ± 0.75	0.81 ± 0.41
Acetyl-CoA carboxylase 2 (*Acacb*)	1.00 ± 0.24	0.95 ± 0.16
Fatty acid synthase (*Fasn*)	1.00 ± 0.91	1.40 ± 1.62

2-month-old C57BL/6J mice were fed a CD for 10 weeks. During the last 4 weeks mice were treated with *Mat1a* antisense oligonucleotide (ASO) (25 mg/kg/week) or with control ASO (25 mg/kg/week) until sacrifice. Acetyl-CoA carboxylase 1 (*Acaca*) (n=6/group), acetyl-CoA carboxylase 2 (*Acacb*) (n=5 for control ASO and 7 for *Mat1a* ASO) and fatty acid shyntase (*Fasn*) (n=7 for control ASO and 6 for *Mat1a* ASO) expression was normalized with glyceraldehydes-3-phosphate dehydrogenase (*Gapdh*) expression. Values are presented as means ± SD of fold change respect to control ASO HFD-fed mice (arbitrary units). Source data are provided as a Source data file.

Supplementary Table 7. Primary antibodies used for Western Blotting analysis (WB), immunohystochemistry (IHQ) and Chromatin immunoprecipitation (Chip). CST; Cell Signaling Technology. R&D; R&D Systems.

Target	Name	Manufacturer	Catalog No.	Concentration/ Quantity	Clone number	Utility
MATI/III	Methionine adenosyltransferase 1 alpha	NOVUS Biologicals	NBP1-55120	1:1000	-	WB
UCP1	Uncoupling protein 1	Abcam	ab10983	1:2000	-	WB
ATF4	Activating transcription factor 4	CST	11815	1:1000	D4B8	WB
NRF2	Nuclear factor erythroid 2-related factor 2	CST	12721	1:1000	D1Z9C	WB
ACC	Acetyl-CoA carboxylase	CST	3662	1:1000	-	WB
p-ACC	Phospho Acetyl-CoA carboxylase	CST	3661S	1:1000	-	WB
FAS	Fatty acid synthase	CST	3189	1:1000	-	WB
PPARα	Peroxisome proliferator activated receptor alpha	Santa Cruz Biotechnology	sc-398394	1:1000	H-2	WB
PGC1a	Peroxisome proliferator activated receptor gamma coactivator 1 alpha	Abcam	ab54481	1:1000	-	WB
CD36	Scavenger Receptor 36	Abcam	ab23680	1:1000	JC63.1	WB
LPL	Lipoprotein Lipase	Santa Cruz Biotechnology	sc-373759	1:1000	F-1	WB
p38	Mitogen Activated Protein Kinase p38	CST	2308S	1:1000	10A8	WB
p-p38	Phospho- Mitogen Activated Protein Kinase p38	CST	9211S	1:1000	-	WB
S6	Ribosomal Protein S6	CST	2317S	1:1000	54D2	WB
p-S6	Phospho-Ribosomal Protein S6	CST	4857S	1:1000	91B2	WB
PKA	Protein Kinase A	CST	4782S	1:1000	-	WB
p-PKA	Phospho-Protein Kinase A	CST	4781S	1:1000	-	WB
β-Klotho	Beta Klotho	R&D	AF2619	1:1000	-	WB
FAB fragment	FAB fragments anti mouse-IgG	Jackson ImmunoResearch Europe, Ltd	115-007-003	1:50	-	IHQ
F4/80	F4/80 (Biotinylated)	Bio-Rad Laboratories Inc	MCA497BB	1:50	Cl:A3-1	IHQ
SUMO1	small ubiquitin-related modifier-1	Abcam	ab32058	1:1000	Y299	WB
Tubulin	Tubulin	Sigma	T4026	1:1000	TUB2.1	WB
Transferrin	Transferrin	Santa Cruz Biotechnology	sc-22597	1:1000	-	WB
GAPDH	Glyceraldehyde 3 phosphate dehydrogenase	Abcam	ab8245	1:20000	0411	WB
Histone H3	Histone H3	CST	9715	1:1000	-	WB
NRF2	Nuclear factor erythroid 2-related factor 2	CST	12721	1 μg per sample	D1Z9C	Chip, WB

Supplementary Table 8. Oligonucleotides and sequences used for quantitative PCR analysis of mouse gene expression. All oligonucleotides were individually designed. All the oligonucleotides were provided by Invitrogen.

Gene	Name	Forward Primer	Reverse Primer
Mat1a	Methionine adenosyltransferase 1 alpha	GACACCATCAAGCACATTGG	ATGCATTCCTCGGTCTCATC
Fgf21	Fibroblast growth factor 21	GGGAGGATGGAACAGTGGTA	GCTTTGACACCCAGGATTTG
Cyp4a14	Cytochrome P450 4 a 14	GGCAGTGTTCAGTTGGATGA	GGCGAAAGAAAGTCAGGTTG
Cyp2b9	Cytochrome P450 2 b 9	CCCTGTTGCTCCAAAGGACA	GGGTGTGAGCAGCTACCAAT
Nqo1	NAD(P)H dehydrogenase [Quinone] 1	TTCTGTGGCTTCCAGGTCTT	CGTTTCTTCCATCCTTCCAG
Cgl	Glutamate-cysteine ligase	AGCGATTACACCACAAACCAA	TCCAATGTCAGCCAACTTCA
Gr	Glutathione reductase	GCCAACAAAGAGGAAAAGGTG	AGCATCTCATCACAGCCAATC
Acaca	Acetyl-CoA carboxylase alpha	TGGTGCAGAGGTACCGAAGTG	GTCGTAGTGGCCGTTCTGAAAG
Acacb	Acetyl-CoA carboxylase beta	TGCTAATGGGTTGTCCTTCC	TCTTGATGTGTGCCTGCTTC
Fasn	Fatty Acid Synthase	GATCCTGGAACGAGAACACGAT	AGAGACGTGTCACTCCTGGACTTG
Nrf2	Nuclear factor erythroid 2-related factor 2	CTACTCCCAGGTTGCCCACA	CGACTCATGGTCATCTACAAATGG
Ucp1	Uncoupling Protein 1	ACCTGCCTCTCTCGGAAAC	TGCCACACCTCCAGTCATTA
Prdm16	PR Domain Containing 16	AGGGCAAGAACCATTACACG	TGAGGTCTGGAGGGGAAGTA
Ppargc1a	Peroxisome proliferator activated receptor gamma coactivator 1 alpha	TATGGAGTGACATAGAGTGTGCT	CCACTTCAATCCACCCAGA
Pparg	Peroxisome proliferator activated receptor gamma	CACTCGCATTCCTTTGACATC	CGCACTTTGGTATTCTTGGAG
Ppara	Peroxisome proliferator activated receptor alpha	GCAGTGCCCTGAACATCGAG	CGTCTGATGAGCATGTCACTGTG
Adipoq	Adiponectin	GCCGTTCTCTTCACCTACGA	ACTTGGTCTCCCACCTCCA
ApoC2	Apolipoprotein C2	CTCGGTTCTTCCTGGCTCTA	GGACCTCATTTCCCAACATC
ApoC3	Apoliporpotein C3	GGAGAGGAAGGAAGGGAAGA	TAGATGGCTGGGTGGTGAG
Bhmt	Betaine--Homocysteine S-Methyltransferase	CGGAGAAGTTGTGATTGGAGA	GCCTTTACATAGCCCCTCTTTT
Sahh	S-adenosylhomocysteine hydrolase	ATTCTGGATGATGGTGGTGAC	GTGGGTATTTGGTGTGGATGA
Cbs	Cystathionine Beta-Synthase	CGGTGGTGGATAAGTGGTTC	AGTCCTTCCTGTGCGATGAG
Nrf2 - Fgf21 regulatory region	*Nrf2* binding site in *Fgf21* regulatory region	CATCAGAAGTCAGCCATCCA	AAGCATTCCAGCATTTCCAG
Actb	Actin	ATCGCTGACAGGATGCAGAAG	TCAGGAGGAGCAATGATCTTGA
Gapdh	Glyceraldehyde 3 phosphate dehydrogenase	TATGACTCCACTCACGGCAAATT	TCGCTCCTGGAAGATGGTGAT

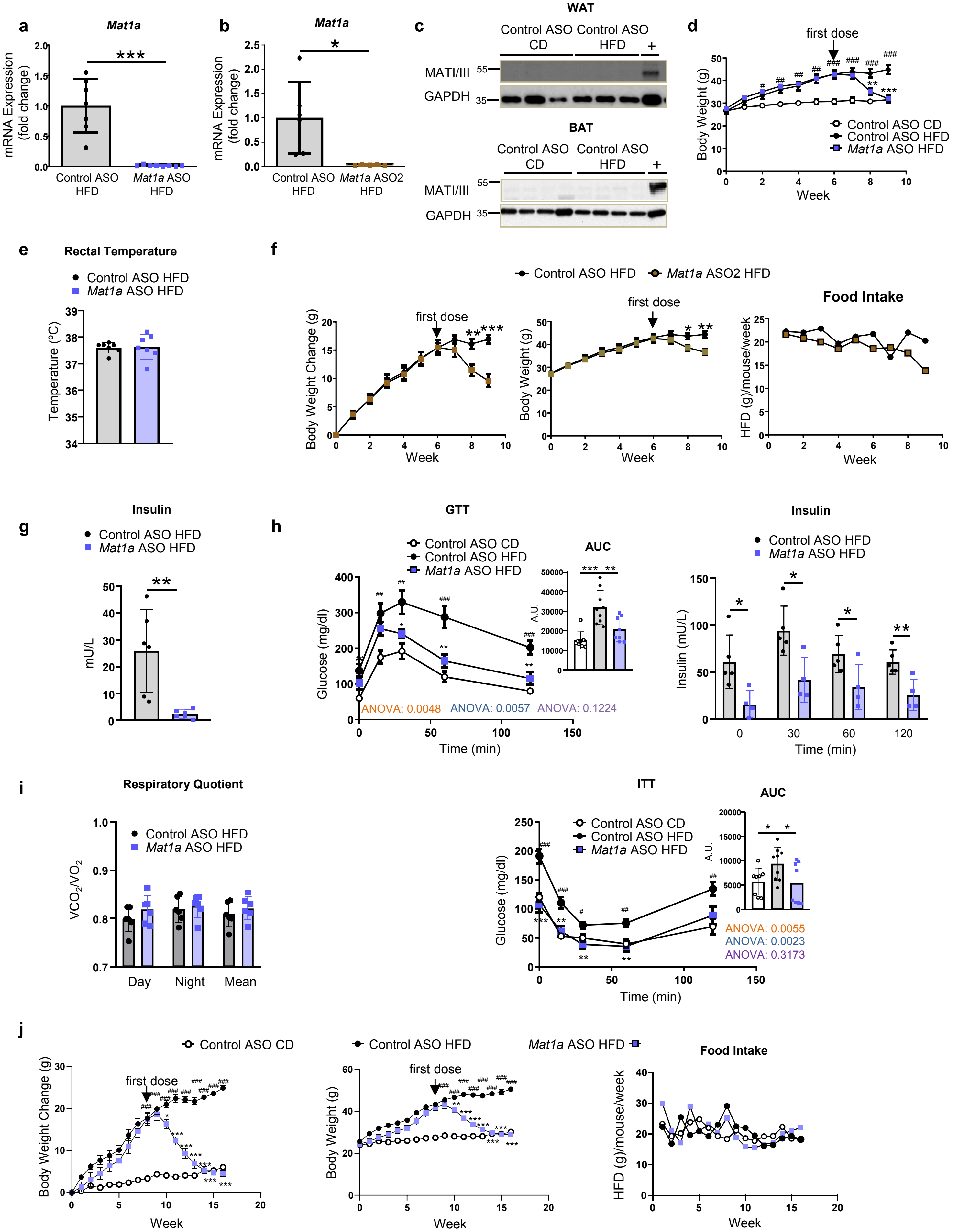
a
Mat1a
***
mRNA Expression (fold change)
Control ASO HFD
Mat1a ASO HFD
b
Mat1a
*
mRNA Expression (fold change)
Control ASO HFD
Mat1a ASO2 HFD
c
WAT
Control ASO CD
Control ASO HFD
+
MATI/III
GAPDH
BAT
d
first dose
Body Weight (g)
Week
Control ASO CD
Control ASO HFD
Mat1a ASO HFD
e
Rectal Temperature
Control ASO HFD
Mat1a ASO HFD
Temperature (ºC)
f
Control ASO HFD
Mat1a ASO2 HFD
first dose
Body Weight Change (g)
Week
Body Weight (g)
Food Intake
HFD (g)/mouse/week
g
Insulin
Control ASO HFD
Mat1a ASO HFD
**
mU/L
h
GTT
Control ASO CD
Control ASO HFD
Mat1a ASO HFD
AUC
A.U.
Glucose (mg/dl)
ANOVA: 0.0048
ANOVA: 0.0057
ANOVA: 0.1224
Time (min)
Insulin
Control ASO HFD
Mat1a ASO HFD
Insulin (mU/L)
Time (min)
i
Respiratory Quotient
Control ASO HFD
Mat1a ASO HFD
VCO_2/VO_2
Day
Night
Mean
ITT
Control ASO CD
Control ASO HFD
Mat1a ASO HFD
AUC
A.U.
Glucose (mg/dl)
ANOVA: 0.0055
ANOVA: 0.0023
ANOVA: 0.3173
Time (min)
j
Control ASO CD
Control ASO HFD
Mat1a ASO HFD
first dose
Body Weight Change (g)
Week
Body Weight (g)
Food Intake
HFD (g)/mouse/week

Supplementary Figure 1. Deficiency in *Mat1a* improves high-fat diet (HFD)-induced obesity. 2-month-old C57BL/6J mice fed chow diet (CD) or HFD, were treated with *Mat1a* antisense oligonucleotides (ASO) (25 mg/kg/week), *Mat1a* ASO2 (50 mg/kg/week), or control ASO (25 and 50 mg/kg/week) until sacrifice. *Mat1a* mRNA expression normalized with Actin (*Actb*) and glyceraldehyde-3-phosphate dehydrogenase (*Gapdh*) in liver of **(a)** HFD-fed control (n=7) and *Mat1a* (n=8) ASO-treated mice and **(b)** control (n=6) and *Mat1a* ASO2 (n=5)-treated mice. **(c)** Blots of MATI/III and GAPDH in white- (WAT) (n=4) and brown-adipose tissues (BAT) (n=4) of HFD-fed ASO-treated mice. **(d)** Body weight of CD- (n=6) and HFD-fed control (n=7) and *Mat1a* (n=8) ASO-treated mice. **(e)** Rectal temperature of HFD-fed control (n=7) and *Mat1a* (n=7) ASO-treated mice. **(f)** Body weight of HFD-fed control (n=6) and *Mat1a* (n=5) ASO2-treated mice. Food intake (n=1 cage). **(g)** Serum insulin levels of HFD-fed control (n=6) and *Mat1a* (n=6) ASO-treated mice fasted overnight. **(h)** Glucose tolerance test (GTT) of CD- (n=8) and HFD-fed control (n=9) and *Mat1a* (n=8) ASO-treated mice at the first week of treatment; insulin serum levels during the GTT of HFD-fed control (n=5) and *Mat1a* (n=4) ASO-treated mice; and insulin tolerance test (ITT) of CD- (n=8) and HFD-fed control (n=9) and *Mat1a* (n=8) ASO-treated mice. Data as area under the curve (AUC). **(i)** Respiratory Quotient of HFD-fed control (n=6) and *Mat1a* (n=6) ASO-treated mice. **(j)** Body weight change and body weight of CD- (n=5) and HFD-fed control (n=5) and *Mat1a* (n=5) ASO-treated mice. Food intake during 16 weeks CD- (n=2 cages) and HFD-fed (n=4 cages/group) mice ASO-treated for 9 weeks. Values are means ± SEM for time courses, and means ± SD for histograms. Statistically significant differences: *p<0.05, **p<0.01, and ***p<0.001 when comparing Control vs. *Mat1a* ASO HFD; and $^{\#}$p<0.05, $^{\#\#}$p<0.01, and $^{\#\#\#}$p<0.001 when comparing Control ASO CD vs HFD (two tailed Student's test). Statistical analysis performed by two-way ANOVA test comparing Control ASO CD vs Control ASO HFD; Control ASO HFD vs. *Mat1a* ASO HFD; Control ASO CD vs. *Mat1a* ASO HFD is presented in GTT and ITT curves. Source data are provided as a Source data file.

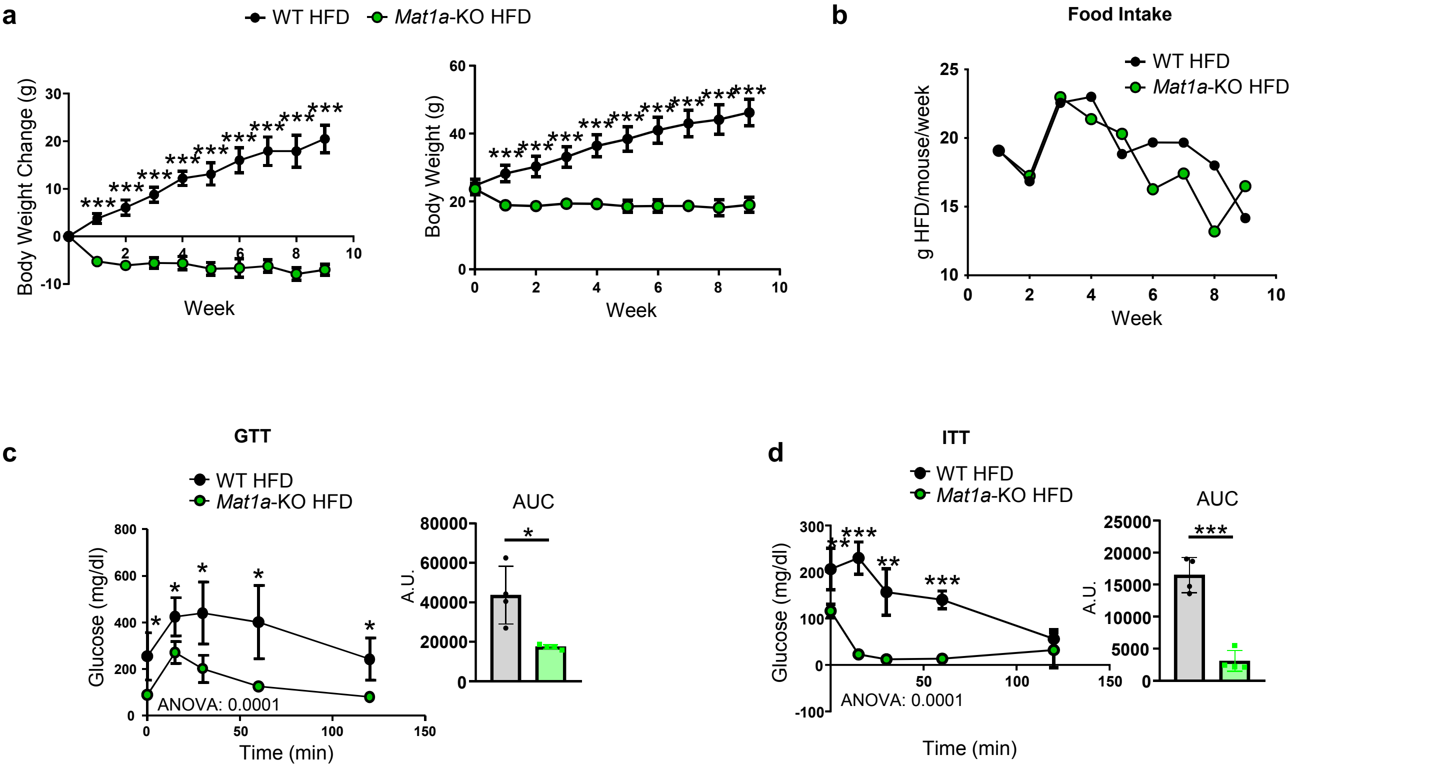

Supplementary Figure 2. Chronic deficiency in *Mat1a* protects from high-fat diet (HFD)-induced obesity. 2-month-old *Mat1a*-KO and WT mice were fed HFD for 10 weeks. **(a)** Body weight change and body weight for HFD-fed WT (n=5) and *Mat1a*-KO (n=4) mice. **(b)** Food intake for HFD-fed WT and *Mat1a*-KO mice (n=2 cages). **(c)** Glucose (GTT) and **(d)** insulin tolerance tests (ITT) for HFD-fed WT (n=4) and *Mat1a*-KO (n=4) mice. Data are also indicated as area under the curve (AUC) expressed in arbitrary units (A.U.). Values are presented as means ± SEM for time course representations, and as means ± SD for histograms. Statistically significant differences between groups are indicated by *$p<0.05$, **$p<0.01$, and ***$p<0.001$ (two tailed Student's test). Statistical analysis performed by two-way ANOVA test is presented in GTT and ITT curves. Source data are provided as a Source data file.

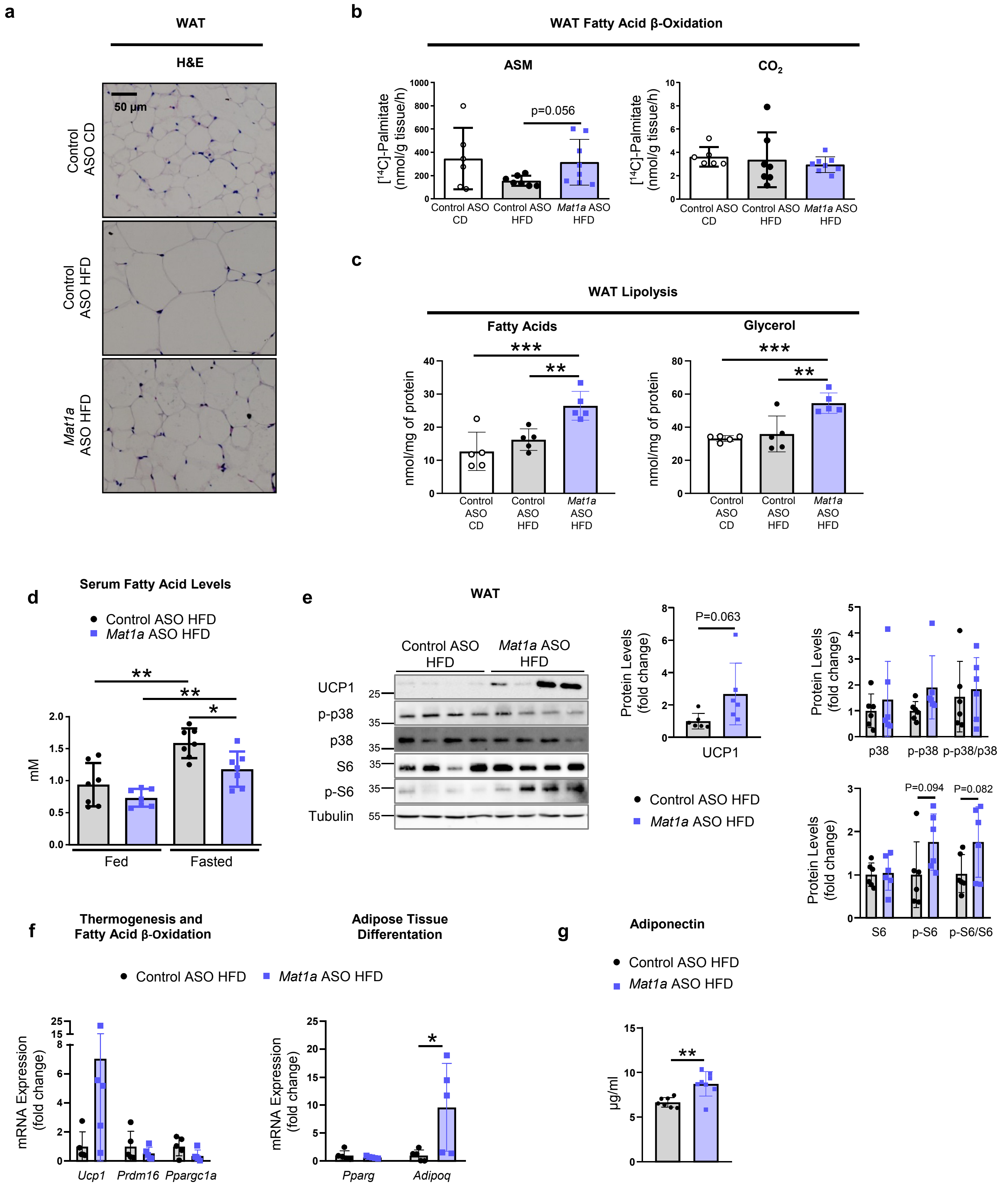
a
WAT
H&E
50 µm
Control ASO CD
Control ASO HFD
Mat1a ASO HFD
b
WAT Fatty Acid β-Oxidation
ASM
CO2
[14C]-Palmitate (nmol/g tissue/h)
p=0.056
Control ASO CD
Control ASO HFD
Mat1a ASO HFD
c
WAT Lipolysis
Fatty Acids
Glycerol
nmol/mg of protein
d
Serum Fatty Acid Levels
Control ASO HFD
Mat1a ASO HFD
mM
Fed
Fasted
e
WAT
UCP1
p-p38
p38
S6
p-S6
Tubulin
Protein Levels (fold change)
P=0.063
P=0.094
P=0.082
p38
p-p38
p-p38/p38
S6
p-S6
p-S6/S6
f
Thermogenesis and Fatty Acid β-Oxidation
Adipose Tissue Differentiation
mRNA Expression (fold change)
Ucp1
Prdm16
Ppargc1a
Pparg
Adipoq
g
Adiponectin
µg/ml

Supplementary Figure 3. Targeting *Mat1a* during high-fat diet (HFD)-induced obesity induces lipolysis in WAT. 2-month-old C57BL/6J mice were fed a chow diet (CD) or a high-fat diet (HFD) for 10 weeks. During the last 4 weeks, mice were treated with *Mat1a* antisense oligonucleotides (ASO) or with control ASO (25 mg/kg/week) until sacrifice. **(a)** Representative microphotographs of CD- and HFD-fed ASO-treated mice (n=6/group) WAT sections stained for hematoxylin-eosin (H&E). **(b)** Fatty acid β-oxidation was determined measuring the amount of [^{14}C]-acid-soluble metabolites (ASM) (incomplete oxidation of palmitate) and [^{14}C]-CO_2 (complete oxidation of palmitate) in WAT from CD- (n=6) and HFD-fed control (n=7) and *Mat1a* (n=8) ASO-treated mice. **(c)** WAT lipolysis was determined by measuring the amount of fatty acids and glycerol secreted *ex vivo* by WAT of CD (n=5) and HFD-fed control (n=5) and *Mat1a* (n=5) ASO-treated mice. **(d)** Serum fatty acid levels in fed and fasted conditions in HFD-fed control (n=7) and *Mat1a* (n=7) ASO-treated mice. **(e)** Representative blots and densitometries of uncoupling protein 1 (UCP1), total and phosphorylated mitogen activated protein kinase p38, total and phsophorylated protein S6 and Tubulin, as representative loading control, in WAT from HFD-fed control (n=6) and *Mat1a* (n=6) ASO-treated mice. **(f)** mRNA expression levels of *Ucp1*, *Prdm16* and *Ppargc1a* and *Pparg* and *Adipoq* in WAT from HFD-fed control (n=5) and *Mat1a* (n=5) ASO-treated mice. **(g)** Serum adiponectin levels in HFD-fed control (n=7) and *Mat1a* (n=8) ASO-treated mice. Values are presented as means ± SD. Statistically significant differences between groups are indicated by *p<0.05, **p<0.01, and ***p<0.001 (two tailed Student's test). Source data are provided as a Source data file.

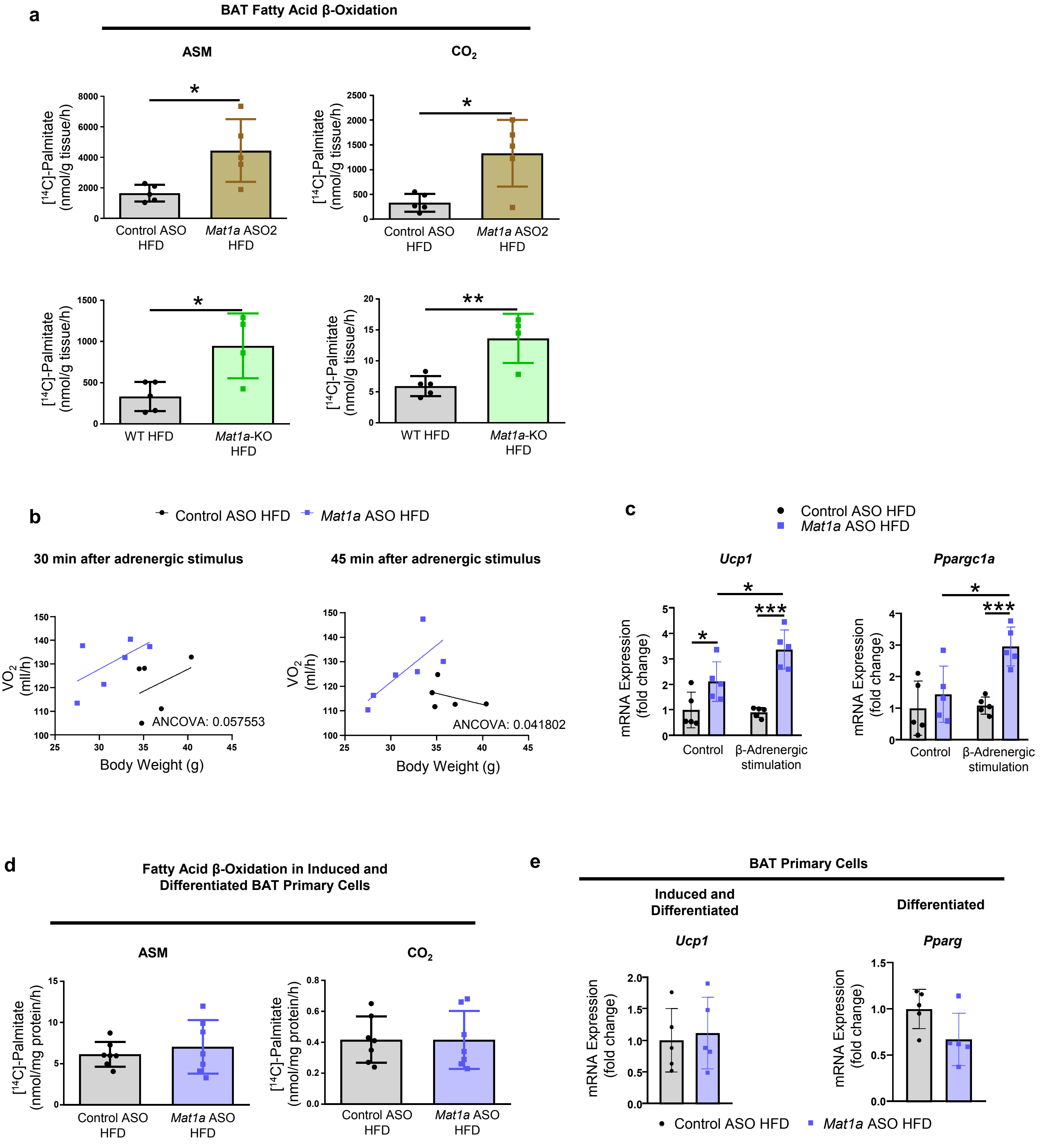

a
BAT Fatty Acid β-Oxidation
ASM
CO2
[14C]-Palmitate (nmol/g tissue/h)
Control ASO HFD
Mat1a ASO2 HFD
WT HFD
Mat1a-KO HFD
*
**
b
Control ASO HFD
Mat1a ASO HFD
30 min after adrenergic stimulus
45 min after adrenergic stimulus
VO2 (ml/h)
Body Weight (g)
ANCOVA: 0.057553
ANCOVA: 0.041802
c
Control ASO HFD
Mat1a ASO HFD
Ucp1
Ppargc1a
mRNA Expression (fold change)
Control
β-Adrenergic stimulation
***
d
Fatty Acid β-Oxidation in Induced and Differentiated BAT Primary Cells
ASM
CO2
[14C]-Palmitate (nmol/mg protein/h)
Control ASO HFD
Mat1a ASO HFD
e
BAT Primary Cells
Induced and Differentiated
Differentiated
Ucp1
Pparg
mRNA Expression (fold change)
Control ASO HFD
Mat1a ASO HFD

Supplementary Figure 4. Targeting liver *Mat1a* increases fatty acid oxidation in brown adipose tissue (BAT). 2-month-old C57BL/6J (C57), WT and *Mat1a*-KO mice were fed a chow diet (CD) or a high-fat diet (HFD) for 10 weeks. During the last 4 weeks, C57 mice were treated with two different *Mat1a* antisense oligonucleotides (ASO), *Mat1a* ASO (25 mg/kg/week) or *Mat1a* ASO2 (50 mg/kg/week) or with control ASO (25 and 50 mg/kg/week) until sacrifice. BAT primary cells were obtained from three independent HFD-fed ASO-treted C57 mice after isolation, growth in culture, induction in some cases and differentiation of adipocyte precursors. **(a)** BAT fatty acid β-oxidation was determined measuring the amount of [^{14}C]-acid-soluble metabolites (ASM) (incomplete oxidation of palmitate) and [^{14}C]-CO_2 (complete oxidation of palmitate) in of HFD-fed control (n=5) and *Mat1a* (n=5) ASO2-treated mice and HFD-fed WT (n=5) and *Mat1a*-KO (n=4) mice. **(b)** Oxygen consumption of HFD-fed control (n=5) and *Mat1a* (n=6) ASO-treated C57 mice after 30 or 45 minutes of a β-adrenergic stimulation. **(c)** *Ucp1* and *Ppargc1a* mRNA expression levels in BAT of HFD-fed ASO-treated mice with or without β-adrenergic stimulation (n=5/group). **(d)** Fatty acid β-oxidation was determined measuring the amount of [^{14}C]-ASM and [^{14}C]-CO_2 in induced and differentiated BAT primary cells (n=7/group) from HFD-fed ASO-treated mice. **(e)** *Ucp1* and *Pparg* mRNA expression levels in induced and differentiated and only differentiated BAT primary cells (n=5/group). Values are presented as means ± SD. Statistically significant differences between groups are indicated by *p<0.05, **p<0.01, and ***p<0.001 (two tailed Student's test). Statistical analysis performed by two-way ANCOVA test is presented in oxygen consumption graphs. Source data are provided as a Source data file.

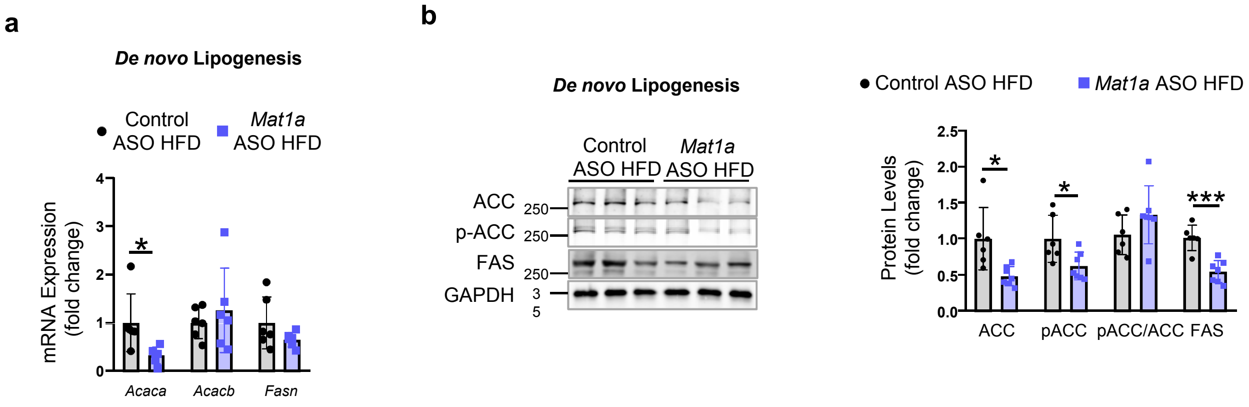

Supplementary Figure 5. Targeting liver *Mat1a* during high-fat diet (HFD)-induced obesity decreases levels of proteins involved in the *de novo* lipogenesis in BAT. 2-month-old C57BL/6J mice were fed a high-fat diet (HFD) for 10 weeks. During the last 4 weeks, mice were treated with *Mat1a* antisense oligonucleotides (ASO) or with control ASO (25 mg/kg/week) until sacrifice. **(a)** Acetyl-CoA carboxylase (ACC) 1 (*Acaca*), ACC2 (*Acacb*) and fatty acid synthase (*Fasn*) mRNA expression levels in BAT from HFD-fed control (n=6) and *Mat1a* (n=6) ASO-treated mice. Results were normalized with actin (*Actb*). **(b)** Representative blots and densitometries of total and phosphorylated ACC, FAS and GAPDH in BAT from HFD-fed control (n=6) and *Mat1a* (n=7) ASO-treated mice. The ratio between phosphorylated and total ACC was also determined. Values are presented as means ± SD. Statistically significant differences between groups are indicated by *p<0.05 and ***p<0.001 (two tailed Student's test). Source data are provided as a Source data file.

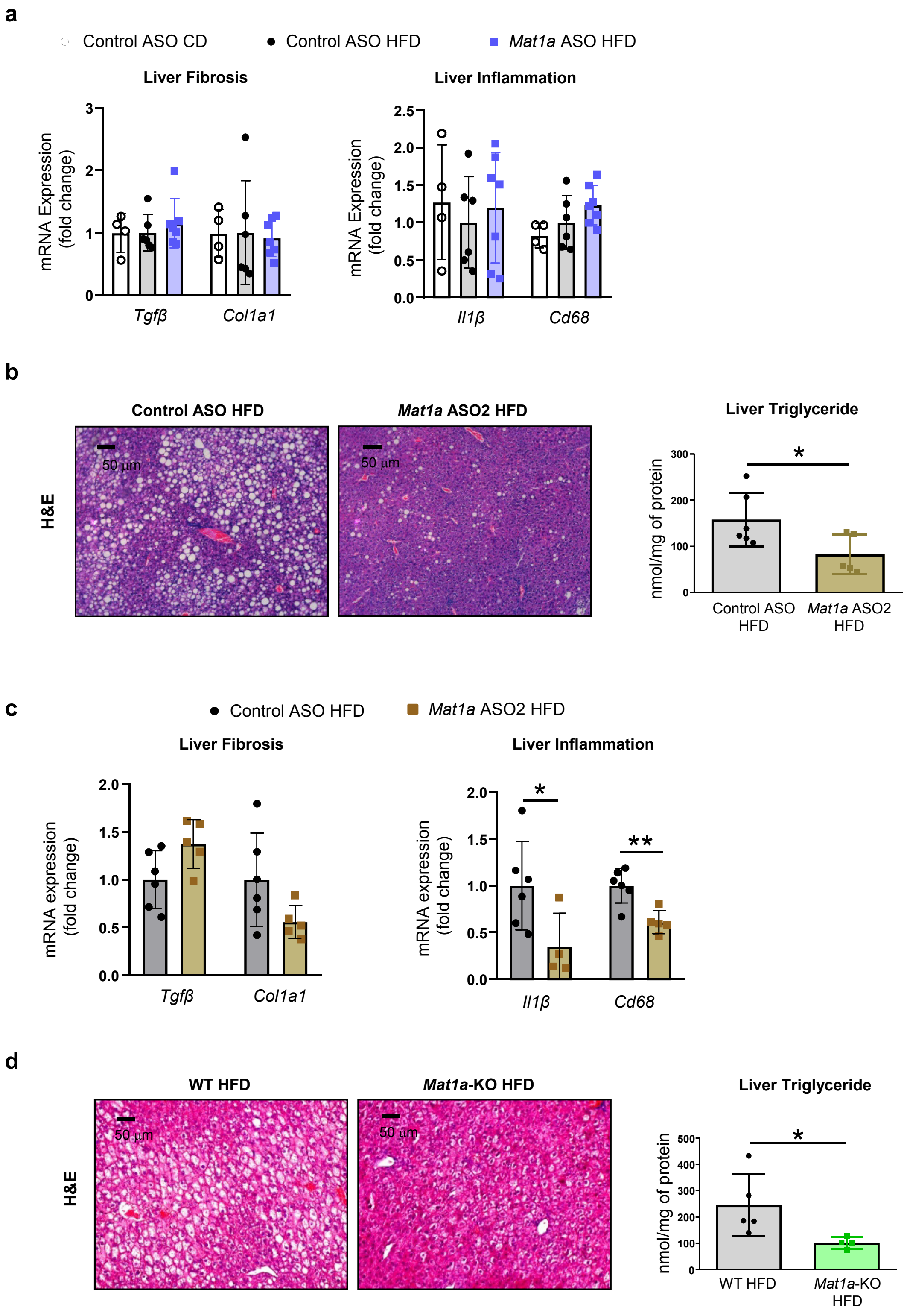
a
Control ASO CD
Control ASO HFD
Mat1a ASO HFD
Liver Fibrosis
Liver Inflammation
mRNA Expression (fold change)
Tgfβ
Col1a1
Il1β
Cd68
b
Control ASO HFD
Mat1a ASO2 HFD
H&E
50 µm
Liver Triglyceride
nmol/mg of protein
*
Control ASO HFD
Mat1a ASO2 HFD
c
Control ASO HFD
Mat1a ASO2 HFD
Liver Fibrosis
Liver Inflammation
mRNA expression (fold change)
Tgfβ
Col1a1
Il1β
Cd68
*
**
d
WT HFD
Mat1a-KO HFD
H&E
50 µm
Liver Triglyceride
nmol/mg of protein
*
WT HFD
Mat1a-KO HFD

Supplementary Figure 6. ***Mat1a*** **deficiency prevents from HFD-induced liver triglyceride storage** 2-month-old C57BL/6J (C57), WT and *Mat1a*-KO mice were fed a high-fat diet (HFD) for 10 weeks. During the last 4 weeks, C57 mice were treated with *Mat1a* antisense oligonucleotides (ASO) (25 mg/kg/week), *Mat1a* ASO2 (50 mg/kg/week), or with control ASO (25 and 50 mg/kg/week) until sacrifice. **(a)** Transforming growth factor beta (*Tgfβ)* and collagen type 1 alpha 1 (*Col1a1),* as liver fibrosis markers, and interleukin 1 beta (*Il1β)* and cluster of differentiation 68 (*Cd68)*, as liver inflammation markers, mRNA expression levels in CD- (n=4) and HFD-fed control (n=6) and *Mat1a* (n=7) ASO-treated mice. Results were normalized with actin (*Actb*). **(b)** Representative liver sections stained with he,atoxylin-eosin (H&E) and liver triglyceride (TG) content of HFD-fed control (n=6) and *Mat1a* (n=5) ASO2-treated mice. **(c)** *Tgfβ* and *Col1a1* and *Il1β* and *Cd68* mRNA expression levels in HFD-fed control (n=6) and *Mat1a* (n=5; n=4 for *Il1β*) ASO2-treated mice. Results were normalized with actin (*Actb*). **(d)** Representative liver sections stained with H&E and liver TG content of HFD-fed WT (n=5) and *Mat1a*-KO (n=4) mice. Values are presented as means ± SD. Statistically significant differences between groups are indicated by *p<0.05, and **p<0.01 (two tailed Student's test). Source data are provided as a Source data file.

a

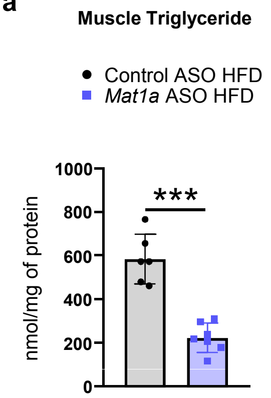

Supplementary Figure 7. *Mat1a* deficiency prevents from HFD-induced muscle triglyceride storage 2-month-old C57BL/6J mice were fed a high-fat diet (HFD) for 10 weeks. During the last 4 weeks, mice were treated with *Mat1a* antisense oligonucleotide (ASO) or with control ASO (25 mg/kg/week) until sacrifice. **(a)** Muscle triglyceride content of HFD-fed control (n=6) and *Mat1a* (n=7) ASO-treated mice. Values are presented as means ± SD. Statistically significant differences between groups are indicated by ***p<0.001 (two tailed Student's test). Source data are provided as a Source data file.

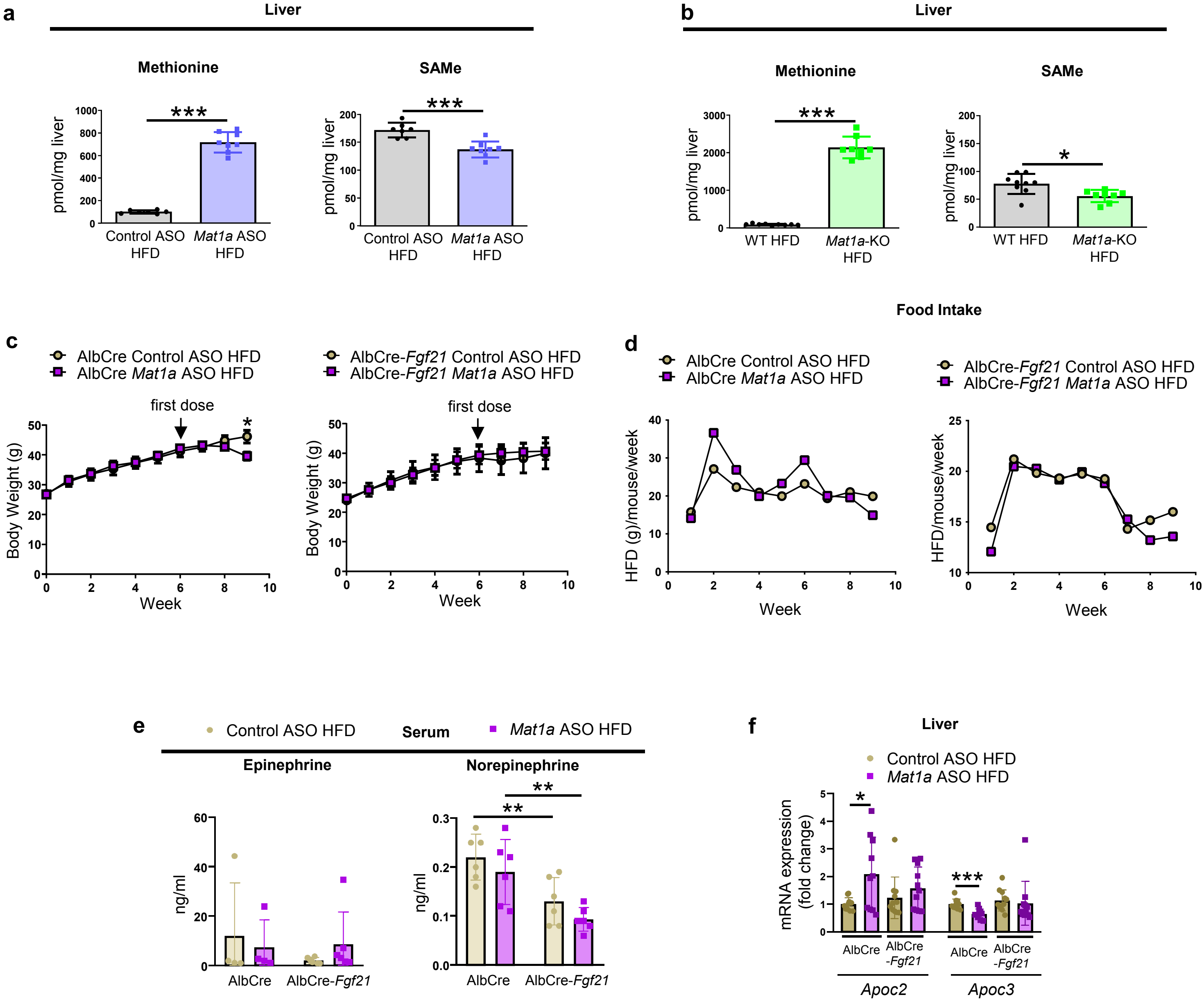

Supplementary Figure 8. Targeting liver *Mat1a* in hepatocyte specific *Fgf21*-KO prevents the altered apoC expression. 2-month-old C57BL/6J (C57), WT, *Mat1a*-KO, AlbCre and AlbCre-*Fgf21* were fed a high-fat diet (HFD) for 10 weeks. During the last 4 weeks, C57, AlbCre and AlbCre-*Fgf21* mice were treated with *Mat1a* antisense oligonucleotides (ASO) or with control ASO (25 mg/kg/week) until sacrifice. **(a)** Liver methionine and S-adenosylmethionine (SAMe) levels of HFD-fed control (n=7) and *Mat1a* (n=8) ASO-treated mice. **(b)** Liver methionine and SAMe levels of HFD-fed WT (n=9) and *Mat1a-KO* (n=8) mice. **(c)** Body weight for HFD-fed control (n=5 and 6) and *Mat1a* (n=5 and 7) ASO-treated AlbCre and AlbCre-*Fgf21* mice, respectively. **(d)** Food intake for HFD-fed control ASO-treated AlbCre and AlbCre-*Fgf21* mice (n=2 cages/group) and *Mat1a* ASO-treated AlbCre (n=2 cages) and AlbCre-*Fgf21* (n=3 cages) mice. **(e)**. Serum epinephrine and norepinephrine levels in HFD-fed control ASO-treated AlbCre (n=4 and 6, respectively) and AlbCre-*Fgf21* (n=4 and 6, respectively) mice and *Mat1a* ASO tretaed AlbCre (n=6/group) and AlbCre-*Fgf21* (n=6/group) mice . **(f)** Liver *Apoc2* and *Apoc3* mRNA expression levels in HFD-fed control (n=10 and 11) and *Mat1a* (n=10 and 11) ASO-treated AlbCre and AlbCre-Fgf21 mice, respectively. Values are presented as means ± SD. Statistically significant differences between groups are indicated by * $p<0.05$, ** $p<0.01$, *** $p<0.001$ (two tailed Student's test). Source data are provided as a Source data file.

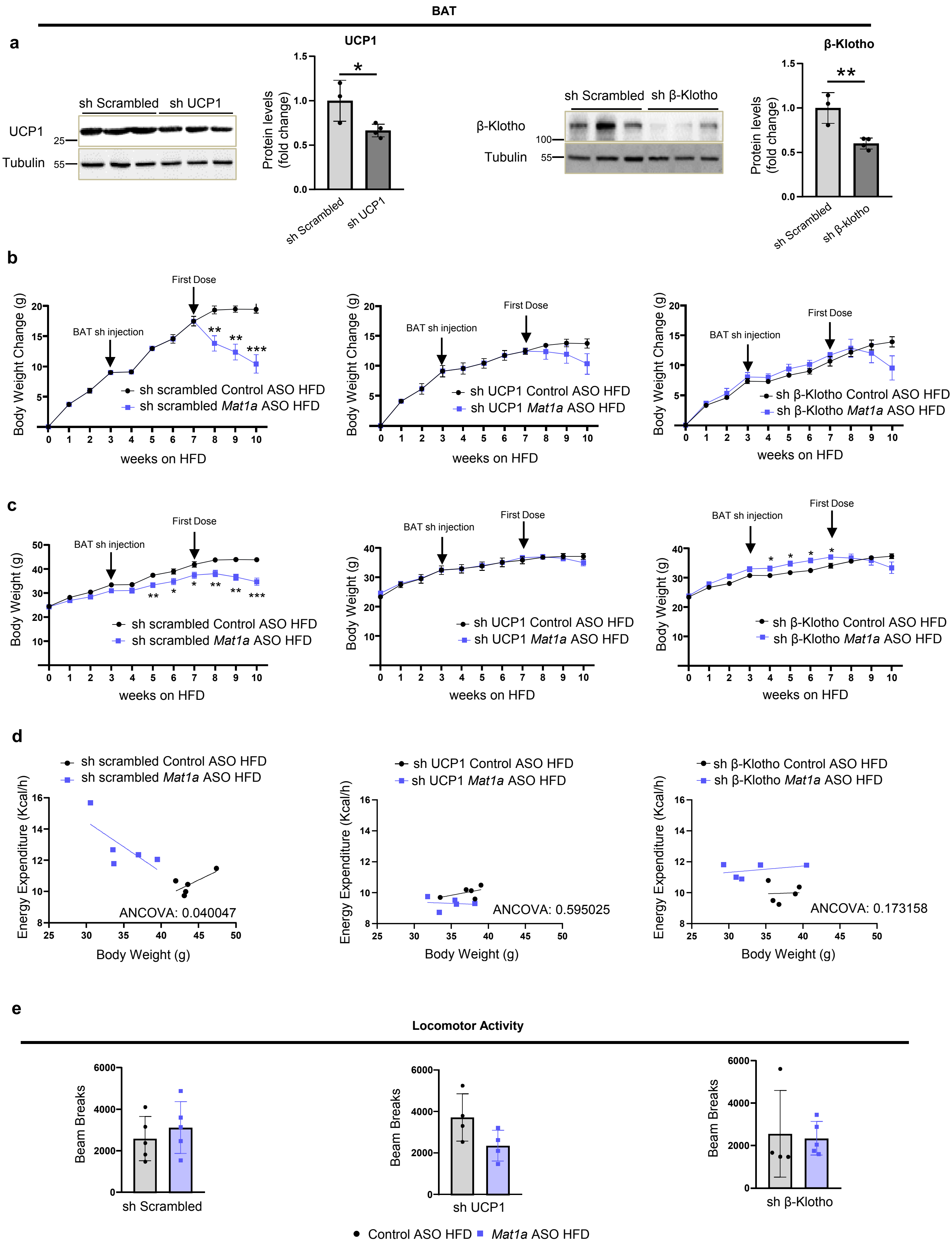

BAT
a
UCP1
sh Scrambled
sh UCP1
Tubulin
25
55
Protein levels (fold change)
*
β-Klotho
sh β-Klotho
100
**
b
Body Weight Change (g)
BAT sh injection
First Dose
sh scrambled Control ASO HFD
sh scrambled Mat1a ASO HFD
sh UCP1 Control ASO HFD
sh UCP1 Mat1a ASO HFD
sh β-Klotho Control ASO HFD
sh β-Klotho Mat1a ASO HFD
weeks on HFD
c
Body Weight (g)
d
Energy Expenditure (Kcal/h)
Body Weight (g)
ANCOVA: 0.040047
ANCOVA: 0.595025
ANCOVA: 0.173158
e
Locomotor Activity
Beam Breaks
sh Scrambled
sh UCP1
sh β-Klotho
Control ASO HFD
Mat1a ASO HFD

Supplementary Figure 9. UCP1 and B-klotho are involved in the *Mat1a* antisense oligonucleotide (ASO) induced brown adipose tissue (BAT) thermogenesis. 2-month-old C57BL/6J mice were treated with lentiviruses containing short hairpin (sh) RNAs against UCP1 (sh UCP1) or against β-Klotho (sh β-Klotho) and fed a high-fat diet (HFD) for 10 weeks. Sh Scrambled were used as control. During the last 4 weeks, mice were treated with a *Mat1a* antisense oligonucleotides (ASO) or with control ASO (25 mg/kg/week) until sacrifice. **(a)** Representative blots and densitometries of UCP1, β-Klotho and tubulin in BAT from HFD-fed mice after lentiviruses treatments (n=3 for sh scrambled, and 4 for sh UCP1 and sh β-Klotho). **(b)** Body weight change in sh Scramble HFD-fed ASO-treated mice, in sh UCP1 HFD-fed ASO-treated mice, and in sh β-Klotho HFD-fed ASO-treated mice (n=5 for each group). **(c)** Body weight in sh Scramble HFD-fed ASO-treated mice, in sh UCP1 HFD-fed ASO-treated mice and in sh β-Klotho HFD-fed ASO-treated mice (n=5 for each group). **(d)** Energy expenditure in sh Scramble HFD-fed ASO-treated mice, in sh UCP1 HFD-fed ASO-treated mice and in sh β-Klotho HFD-fed ASO-treated mice (n=5 for each group). **(e)** Locomotor activity in sh Scrambled (n=5/group), sh UCP1 (n=4/group) and sh β-Klotho (Control ASO HFD n=4; *Mat1a* ASO HFD n=5) HFD-fed ASO-treated mice. Values are presented as means ± SEM for time course representations, and as means ± SD for histograms. Statistically significant differences between groups are indicated by *$p<0.05$, **$p<0.01$, and ***$p<0.001$ (two tailed Student's test). Statistical analysis performed by two-way ANCOVA test is presented in energy expenditure experiments. Source data are provided as a Source data file.

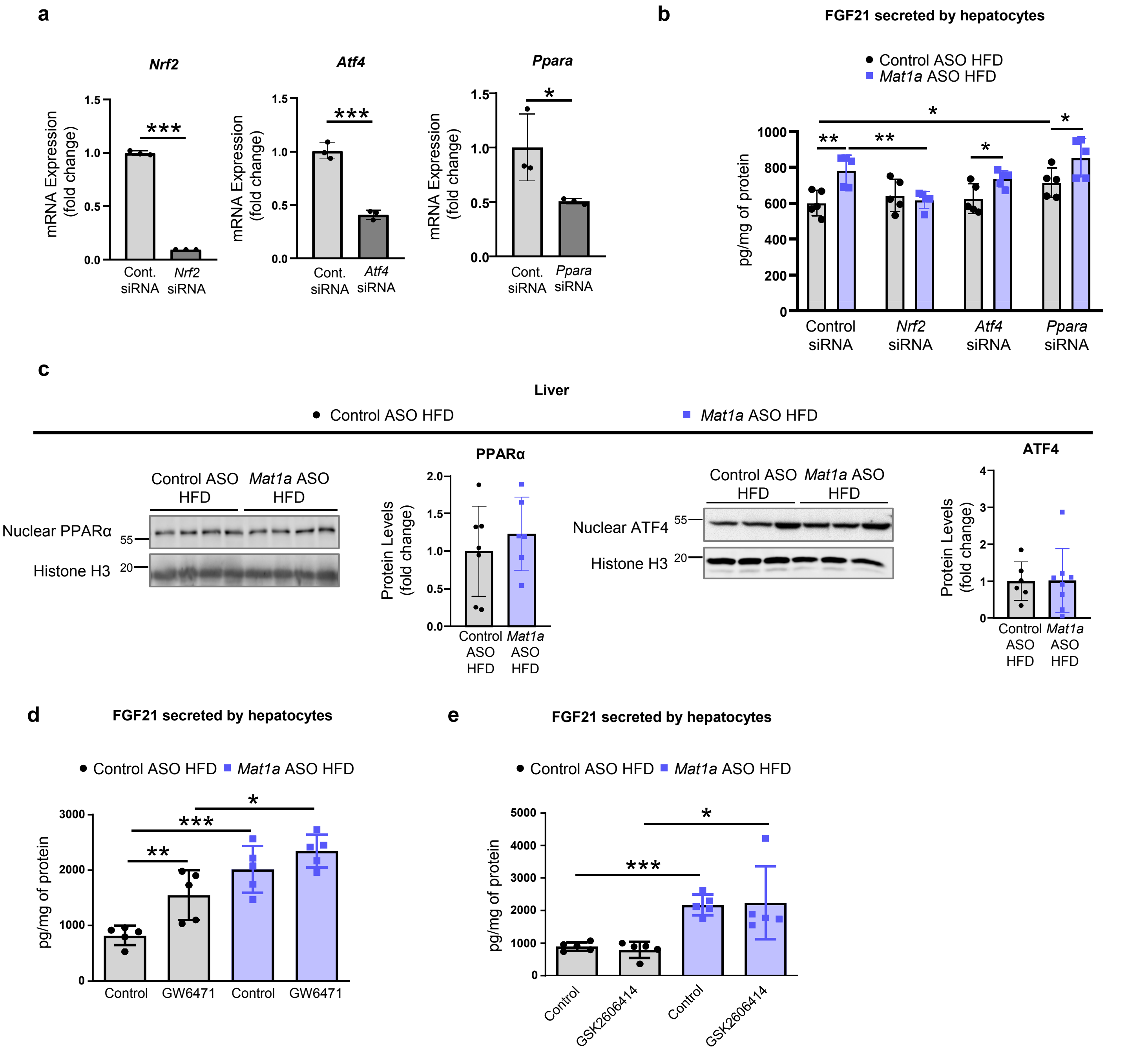

Supplementary Figure 10. *Mat1a* antisense oligonucleotide (ASO)-driven FGF21 increased secretion is not driven by PPARa or by altered unfolded protein response. 2-month-old C57BL/6J mice were fed a high-fat diet (HFD) for 10 weeks. During the last 4 weeks mice were treated with *Mat1a* antisense oligonucleotide (ASO) or with control ASO (25 mg/kg/week) until sacrifice. Hepatocytes from three different HFD-fed ASO-treated mice were isolated, seeded (10^6 or 75000 cells/well) to be treated with siRNAs**.** **(a)** Nuclear factor erythroid 2-related factor 2 (*Nrf2)*, activating transcription factor 4 (*Atf4)* and peroxisome proliferator activated aceeptor alpha (*Ppara*) mRNA expression levels in hepatocytes from HFD-fed ASO-treated mice after siRNAs treatment (n=3/group). **(b)** FGF21 levels secreted by hepatocytes from HFD-fed ASO-treated mice treated with control, *Nrf2*, *Atf4* and *Ppara* siRNAs (n=5/group). **(c)** Representative blots and densitometry of nuclear PPARα, ATF4 and Histone H3 in liver of HFD-fed control (n=7 and 6, respectively) and *Mat1a* (n=6 and 8, respectively) ASO-treated mice. **(d)** FGF21 secreted by hepatocytes from HFD-fed ASO-treated mice exposed to 100 μM of the PPARα inhibitor, GW6471, or vehicle (n=5 for each group). **(e)** FGF21 secreted by hepatocytes from HFD-fed ASO-treated mice exposed to 2 μM of the PERK inhibitor, GSK2606414, or vehicle (n=5 for each group). Values are presented as means ± SD. Statistically significant differences between groups are indicated by *p<0.05, **p<0.01 and ***p<0.001 (two tailed Student's test). Source data are provided as a Source data file.

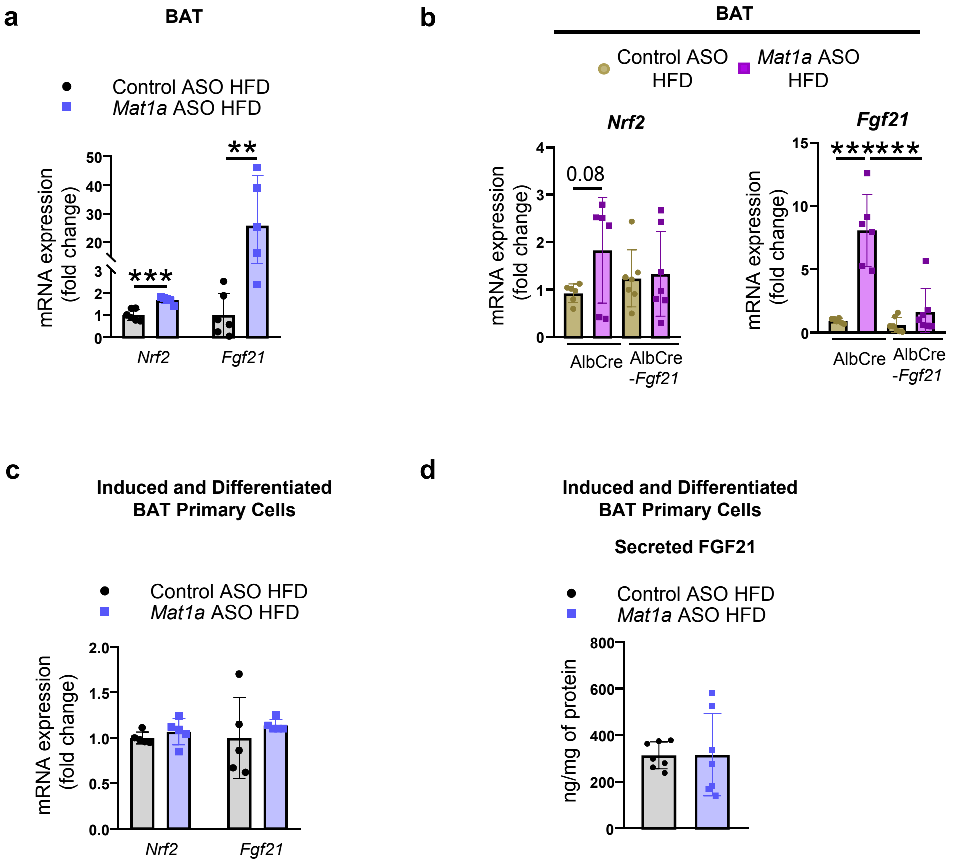

Supplementary Figure 11. Targeting liver *Mat1a* induces expression of fibroblast growth factor 21 (*Fgf*21) in BAT. 2-month-old C57BL/6J (C57), AlbCre and AlbCre-*Fgf21* were fed a high-fat diet (HFD) for 10 weeks. During the last 4 weeks, C57, AlbCre and AlbCre-*Fgf21* mice were treated with *Mat1a* antisense oligonucleotide (ASO) or with control ASO (25 mg/kg/week) until sacrifice. BAT primary cells were obtained from three independent HFD-fed ASO-treted C57 mice after isolation, growth in culture, induction in some cases and differentiation of adipocyte precursors. **(a)** Nuclear factor erythroid 2-related factor 2 (*Nrf2)* and *Fgf21* mRNA expression levels in BAT from HFD-fed control (n=6) and *Mat1a* (n=5) ASO-treated mice. **(b)** *Nrf2* and *Fgf21* mRNA expression levels in BAT from HFD-fed control and *Mat1a* ASO-treated AlbCre (n=6/group) and *AlbCre-Fgf21* (n=7/group) mice, respectively. **(c)** *Nrf2* and *Fgf21* mRNA expression levels in BAT primary cells (n=5/group) isolated from HFD-fed ASO-treated mice. **(d)** FGF21 levels secreted by BAT primary cells (n=7/group) isolated from HFD-fed ASO-treated mice. Values are presented as means ± SD for histograms. Statistically significant differences between groups are indicated by $**p<0.01$, and $***p<0.001$ (two tailed Student's test). Source data are provided as a Source data file.

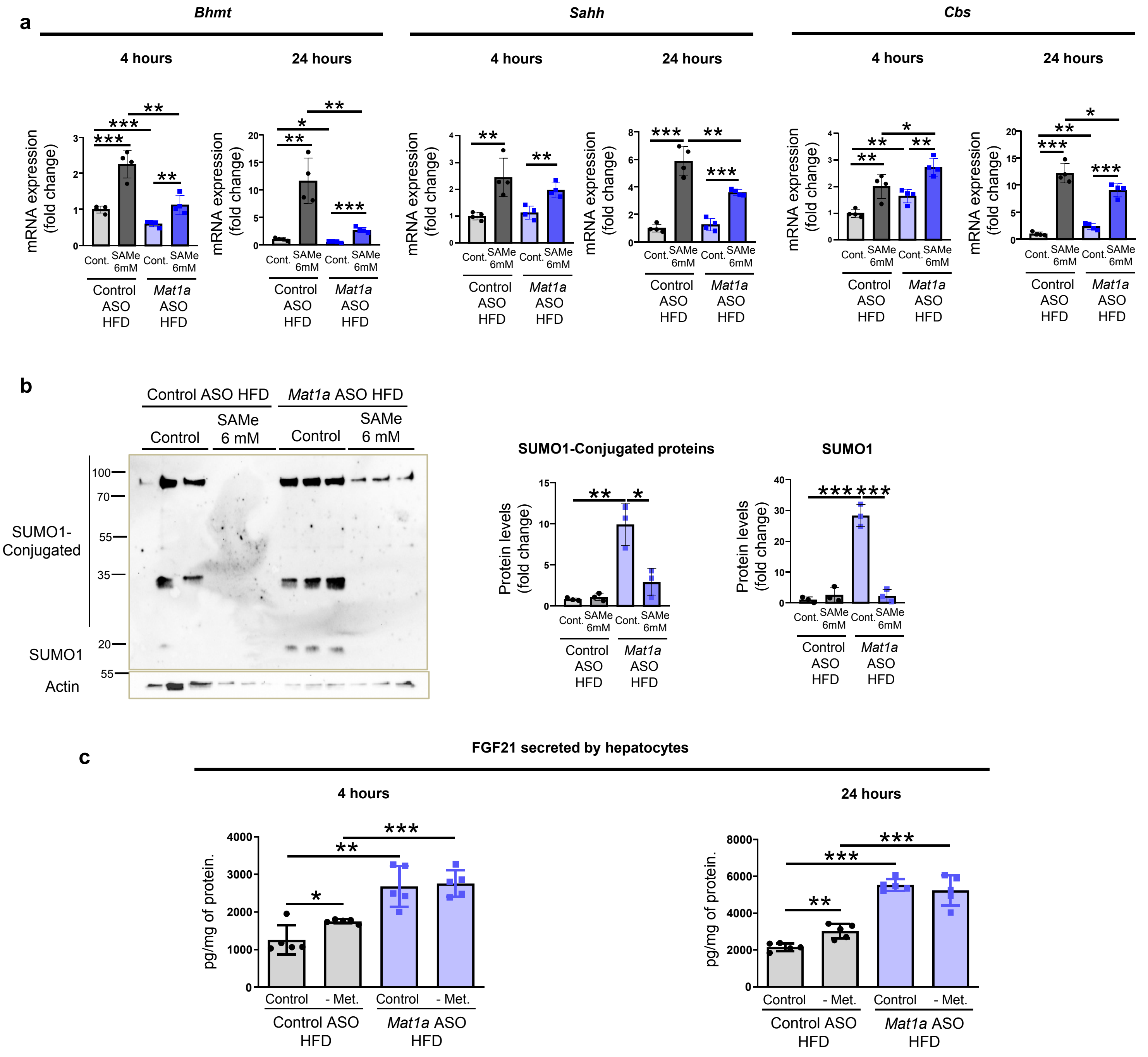

Supplementary Figure 12. Response of hepatocytes isolated from HFD-fed ASO-treated mice to S-adenosylmethionine (SAMe). 2-month-old C57BL/6J mice were fed a high-fat diet (HFD) for 10 weeks. During the last 4 weeks mice were treated with *Mat1a* antisense oligonucleotide (ASO) or control ASO (25 mg/kg/week) until sacrifice. Hepatocytes from three different HFD-fed ASO-tretaed mice were isolated, seeded (10^6 or 75.000 cells/well) to be cultured on a methionine deficient medium or treated with SAMe **(a)** Betaine-homocysteine S-methyltransferase (*Bhmt*), S-adenosylhomocysteine hydrolase (*Sahh)* and cystathionine beta-synthase (*Cbs)* mRNA expression levels in hepatocytes from HFD-fed ASO-treated mice exposed to 6 mM of SAMe for 4 and 24 hours (n=4/group). **(b)** Representative blots and densitometry of small ubiquitin-like modifier (SUMO1)-Conjugated proteins and SUMO1 levels in hepatocytes from HFD-fed ASO-treated mice exposed to 6 mM of SAMe for 24 hours (n=3/group). **(c)** FGF21 secreted by hepatocytes from HFD-fed ASO-treated mice exposed to a methionine defficient medium for 4 hours and 24 hours (n=5/group). Values are presented as means ± SD. Statistically significant differences between groups are indicated by *p<0.05, **p<0.01, and ***p<0.001 (two tailed Student's test). Source data are provided as a Source data file.
