# Supplementary material for: Methionine adenosyltransferase 1a antisense oligonucleotides activate the liver-brown adipose tissue axis preventing obesity and associated hepatosteatosis
Source: Nat Commun. 2022 Mar 1;13:1096. doi: 10.1038/s41467-022-28749-z (PMC8888704; doi:10.1038/s41467-022-28749-z)
Supplement: Supplementary file 3 — Reporting Summary [file 41467_2022_28749_MOESM3_ESM.pdf]

nature portfolio

Corresponding author(s): Patricia Aspichueta

Last updated by author(s): Jan 17, 2022

Reporting Summary

Nature Portfolio wishes to improve the reproducibility of the work that we publish. This form provides structure for consistency and transparency in reporting. For further information on Nature Portfolio policies, see our Editorial Policies and the Editorial Policy Checklist.

Statistics

For all statistical analyses, confirm that the following items are present in the figure legend, table legend, main text, or Methods section.

n/a	Confirmed	
☐	☒	The exact sample size (*n*) for each experimental group/condition, given as a discrete number and unit of measurement
☐	☒	A statement on whether measurements were taken from distinct samples or whether the same sample was measured repeatedly
☐	☒	The statistical test(s) used AND whether they are one- or two-sided<br>*Only common tests should be described solely by name; describe more complex techniques in the Methods section.*
☒	☐	A description of all covariates tested
☒	☐	A description of any assumptions or corrections, such as tests of normality and adjustment for multiple comparisons
☐	☒	A full description of the statistical parameters including central tendency (e.g. means) or other basic estimates (e.g. regression coefficient) AND variation (e.g. standard deviation) or associated estimates of uncertainty (e.g. confidence intervals)
☐	☒	For null hypothesis testing, the test statistic (e.g. *F*, *t*, *r*) with confidence intervals, effect sizes, degrees of freedom and *P* value noted<br>*Give P values as exact values whenever suitable.*
☒	☐	For Bayesian analysis, information on the choice of priors and Markov chain Monte Carlo settings
☒	☐	For hierarchical and complex designs, identification of the appropriate level for tests and full reporting of outcomes
☒	☐	Estimates of effect sizes (e.g. Cohen's *d*, Pearson's *r*), indicating how they were calculated

Our web collection on statistics for biologists contains articles on many of the points above.

Software and code

Policy information about availability of computer code

Data collection	Flir R -Tools specific software package for analysis of corporal and interscapular temperature measurement; FRIDA 1.0 software for histochemistries; Quantity One 29.0 and ImageLab version 6.0.1 softwares for western blotting analysis; INSECT 2.0 software was used to analyze binding sites in gene promoter.
Data analysis	GeNorm 3.1; GraphPad Prism version 8.0 was used to analyse data. The Excel 2016 t-test was also used.

For manuscripts utilizing custom algorithms or software that are central to the research but not yet described in published literature, software must be made available to editors and reviewers. We strongly encourage code deposition in a community repository (e.g. GitHub). See the Nature Portfolio guidelines for submitting code & software for further information.

Data

Policy information about availability of data

All manuscripts must include a data availability statement. This statement should provide the following information, where applicable:

- Accession codes, unique identifiers, or web links for publicly available datasets
- A description of any restrictions on data availability
- For clinical datasets or third party data, please ensure that the statement adheres to our policy

No data sets with mandated depositions are presented in the study. Data supporting the findings of this study are available within the paper and its supplementary information files. Source data are provided with this paper.

Field-specific reporting

Please select the one below that is the best fit for your research. If you are not sure, read the appropriate sections before making your selection.

☒ Life sciences ☐ Behavioural & social sciences ☐ Ecological, evolutionary & environmental sciences

For a reference copy of the document with all sections, see nature.com/documents/nr-reporting-summary-flat.pdf

Life sciences study design

All studies must disclose on these points even when the disclosure is negative.

Sample size	The fewest number of mice were used to achieve statistical significance. Pilot and previous studies on the basis of literature documentation of similar well characterized experiments (papers below) were used to determine the sample size.The sample size and the power to detect the differences between experimental groups are provided in figure legends and in the Statistical Analysis section in Methods.<br><br>Porteiro, B., Fondevila, M., Delgado, T. et al. Hepatic p63 regulates steatosis via IKK!/ER stress. Nat Commun 8, 15111 (2017). https://doi.org/10.1038/ ncomms15111<br><br>González-Romero F, Mestre D, Aurrekoetxea I, et al. E2F1 and E2F2-Mediated Repression of CPT2 Establishes a Lipid-Rich Tumor-Promoting Environment. Cancer Res. 81 (11): 2874-2887. (2021) doi: 10.1158/0008-5472.CAN-20-2052.
Data exclusions	In the locomotor activity of mice in which Ucp1 and b-klotho were silenced, one outlier has not been taken into consideration for the analysis. It is marked in red in the excel document that has been uploaded. The exclusion criteria was pre-established. The samples were excluded because the value was outside the 2SD range.
Replication	Multiple independent experiments were conducted to verify the reproducibility of the data. For this study 310 mice have been used, from those 293 mice were treated with the ASOs (152 with control ASO, 126 with Mat1a ASO and 15 with MAt1a ASO2), the rest were Mat1a-KO mice and their controls. Independent experiments using Mat1a ASO and control ASO have been performed several times, even in different laboratories (Aspichueta Lab-UPV/EHU, Sabio Lab-CNIC, Nogueiras lab-CIMUS). Experiments in vivo were repeated at least ten times and the in vitro experiments were repeated at least three times. All attempts to replicate experiments were successful.
Randomization	Previous to the experiments, all mice groups were made with set of animals of the same sex, age and similar body weight. See methods section. The distribution of the animals with the same genotype to receive the treatments was randomized.
Blinding	The end up experiments were always performed blind; thus, tissues and blood were collected without knowing the group of each mice.

Reporting for specific materials, systems and methods

We require information from authors about some types of materials, experimental systems and methods used in many studies. Here, indicate whether each material, system or method listed is relevant to your study. If you are not sure if a list item applies to your research, read the appropriate section before selecting a response.

Materials & experimental systems

n/a	Involved in the study	
☐	☒	Antibodies
☐	☒	Eukaryotic cell lines
☒	☐	Palaeontology and archaeology
☐	☒	Animals and other organisms
☒	☐	Human research participants
☒	☐	Clinical data
☒	☐	Dual use research of concern

Methods

n/a	Involved in the study	
☒	☐	ChIP-seq
☒	☐	Flow cytometry
☒	☐	MRI-based neuroimaging

Antibodies

Antibodies used	All the antibodies used in this study have been detailed in Supplementary table 3, including supplier, catalog number, dilution and clone. Used antibodies were against:<br>Methionine adenosyltransferase 1 alpha (MATI/III; NOVUS Biologicals; NBP1-55120)<br>Uncoupling protein 1 (UCP1; Abcam; ab10983)<br>Activating factor 4 (ATF4, CST; 11815)<br>Nuclear factor erythoid 2-related factor 2 (NRF2; CST; 12721)<br>Acetyl-CoA carboxylase (ACC; CST; 3662)<br>Phospho acetyl-Co carboxylase (p-ACC; CST; 3661S)<br>Fatty acid synthases (FAS; CST; 3189)<br>Peroxisome proliferator activated receptor alpha (PPARalpha; Sta Cruz Biotech; sc-398394)

Peroxisome proliferator activated receptor gamma coactivator 1 alpha (PGC1a; Abcam; ab54481)
Scavenger receptor 36 (CD36; Abcam; ab23680)
Lipoprotein lipase (LPL; Sta Cruz Biotech; sc-373759)
Mitogen activated protein kinase p38 (p38; CST; 2308S)
Phopho-mitogen activated protein kinase p38 (p-p38; CST; 9211S)
Ribosomal protein s6 (S6; CST; 2317S)
Phospho-ribosomal protein s6 (p-S6; CST; 4857S)
Protein kinase A (PKA; CST; 4782S)
Phospho-protein kinase A (p-PKA; CST; 4781S)
Beta klotho (B-klotho; R&D; AF2619)
FAB fragments anti mouse-IgG (FAB fragment; Jack ImmunoRes Eu; 115-007-003)
F4/80 (F4/80; Bio-Rad Lab; MCA497BB)
Small ubiquitin-related modifier-1 (SUMO1; Abcam; ab32058)
Tubulin (tubulin; sigma; T4026)
Transferrin (transferrin; Sta Cruz biotech; sc-22597)
Glyceraldehide 3 phosphate dehydrogenase (GAPDH; Abcam; ab8245)
Histone H3 (Histone H3; CST; 9715)
Nuclear factor erythroid 2-related factor 2 (NRF2; CST; 12721)

Validation

The validation information can be found online for antibodies from Cell Signaling (https://www.cellsignal.com/about-us/cst-antibodyvalidation-principles), Abcam (https://www.abcam.com/primary-antibodies/howwe-validate-our-antibodies), R&D Systems (https://www.rndsystems.com/products/antibodies) and Santa Cruz (https://www.scbt.com).
This information can also be obtained from external antibody browsers such as biocompare (https://www.biocompare.com/Antibodies/), Citeab (https://www.citeab.com/) and Antibodyresource (https://www.antibodyresource.com/findantibody.html).

Eukaryotic cell lines

Policy information about cell lines

Cell line source(s)

Human kidney ephithelial HEK-293T cells (The Global Bioresource center, CRL-3216) have been authenticated by STR profiling (ATCC).

Authentication

The 293T cell line, originally referred as 293tsA1609neo, is a highly transfectable derivative of human embryonic kidney 293 cells, and contains the SV40 T-antigen. This cell line is competent to replicate vectors carrying the SV40 region of replication. It gives high titers when used to produce retroviruses. It has been widely used for retroviral production, gene expression and protein production.

Mycoplasma contamination

Routine testing to make sure no mycoplasma contamination in the cell culture system is always performed.

Commonly misidentified lines
(See ICLAC register)

HEK293T cell line is listed in the table 1 of the database of commonly misidentified cell lines (ICLAC-00063). However, it has been authenticated by STR profiling (ATCC).

Animals and other organisms

Policy information about studies involving animals; ARRIVE guidelines recommended for reporting animal research

Laboratory animals

10 week-old male C57BL/6J mice and C57BL76J liver specific fibrobalst growth factor 21 (fgf21) knock out (AlbCre-Fgf21) and 12 week-old male B6.Cg-Lepob (ob/ob) mice used for Mat1a gene knockdown in the liver. 10 week-old Mat1a knockout (Mat1a-KO) male mice were also included. Mice were fed a rodent chow diet (Teklad Global 18% Protein Rodent Diet 2018S; Envigo INC., USA) or a high-fat diet (HFD) (60% fat calories, Bioserv. F3282) during 10 weeks. For ob/ob mice, HFD treatment was maintained during the weeks of the ASO treatments. Mice body weight and food intake were measured weekly. All mice were housed in a temperature of 21-22 ºC and 40% humidity-controlled room with a 12 h-light/ dark cycle and ad libitum access to food and water.

Wild animals

No wild animals were used in the study.

Field-collected samples

No field collected samples were used in the study

Ethics oversight

Animal procedures were approved by the Ethics Committee for Animal Welfare of the University of the Basque Country UPV/EHU, CIMUS, University of Santiago de Compostela-Instituto de Investigación Sanitaria, and Centro Nacional de Investigaciones Cardiovasculares (CNIC) and were conducted in conformity with the EU Directives for animal experimentation.

Note that full information on the approval of the study protocol must also be provided in the manuscript.
